# Morphologically Controllable Hierarchical ZnO Microspheres Catalyst and Its Photocatalytic Activity

**DOI:** 10.3390/nano12071124

**Published:** 2022-03-29

**Authors:** Xiaoqian Ai, Shun Yan, Ligang Ma

**Affiliations:** 1 College of Physics and Electronic Engineering, Jiangsu Second Normal University, Nanjing 210013, China; aixiaoqian1987@163.com; 2 School of Electronic Engineering, Nanjing Xiaozhuang University, Nanjing 211171, China; yanshun800212@163.com

**Keywords:** hierarchical ZnO microspheres, photocatalysis, hydrothermal, degradation mechanism

## Abstract

The degradation of pollutants in wastewater using abundant resources and renewable energy sources, such as light, is attractive from an environmental perspective. ZnO is a well-known photocatalytic material. Therefore, in this study, a hierarchical ZnO microsphere precursor was prepared using a hydrothermal method. The precursor was subsequently annealed at different temperatures, which enabled the production of a ZnO catalyst having a controllable morphology. Specifically, as the annealing temperature increased, the precursor crystallized into hexagonal wurtzite and the crystallinity also increased. The catalysts were tested for their photocatalytic activity for the degradation of dye molecules (methylene blue and rhodamine B), and the catalyst sample annealed at 400 °C showed the best photocatalytic activity. The origin of this activity was studied using electron paramagnetic resonance spectroscopy and transient photocurrent measurements, and the structure of the optimal catalyst was invested using electron microscopy measurements, which revealed that it was formed of two-dimensional nanosheets having smooth surfaces, forming a 2D cellular network. Thus, we have presented a promising photocatalyst for the mineralization of organic contaminants in wastewater.

## 1. Introduction

In recent years, the utilization of solar energy has become popular as an environmentally friendly route for energy generation and utilization [1,2]. Solar energy can be exploited using photovoltaic solar cells, which convert solar energy into electric energy [3,4,5] or by using photocatalysts to decompose water to hydrogen and oxygen [6]. Further, on light irradiation, cathode semiconductor catalysts can produce electricity and carry out the catalytic reduction of CO_2_ [7], thus achieving the “double carbon” plan. In addition, micro-to-nano photocatalytic devices can turn seawater into fresh water [8], and the careful design of photocatalyst interfaces can greatly improve chemical reactions efficiency, thus avoiding the use of excessive reactants or reductants [9]. Moreover, solar energy can be used to clean polluted water using photocatalysts [9,10,11].

ZnO is a group II–VI wide-band gap semiconductor (its band gap is 3.4 eV [12]) and can be used for ultraviolet blue light emission. The exciton binding energy of ZnO reaches 60 meV (compared with 21 meV for GaN), enabling near band edge composite luminescence at room or higher temperatures. In addition, ZnO is easy to prepare as large-area samples, as well as via epitaxial growth at low temperatures. Further, ZnO has strong radiation resistance. Therefore, ZnO has applications in many fields, including photocatalysis [13,14], photovoltaics [15], and gas sensing [16,17].

ZnO having unique morphologies can be obtained by various methods [18,19,20]. For example, nanoscale porous ZnO can be prepared using metformin as a template, and this material has excellent photon-to-current conversion efficiency [21]. In addition, ZnO nano-mushrooms can be prepared by the solution combustion method for use in the photocatalytic degradation of methyl orange [22]. In addition, *Panax* extract mediated ZnO nano-flowers have been synthesized and applied for the removal of industrial dies using UV illumination [23], and an oriented hierarchical ZnO flower rod architecture was prepared on indium-doped tin oxide glass to photodegrade the organic dye rhodamine B (RhB). As shown by these examples, the morphology determines the specific surface area of the catalyst and the exposed crystal surfaces [24], which also affects the active sites and determines the catalytic performance [25].

In this study, a hierarchical ZnO microsphere precursor was prepared by hydrothermal synthesis and annealed at different temperatures to obtain ZnO catalysts. The effects of the annealing temperature on the crystallinity and morphology were studied, revealing that the morphology, crystallization quality, and band gap can be controlled by varying the annealing temperature. Then, the photodegradation of RhB and methylene blue (MB), two organic dyes, in aqueous solution in the presence of the photocatalyst under light irradiation was studied. We found that the annealing temperature had a significant effect on the photocatalytic ability of the ZnO microspheres, and the optimum catalyst having a large pore size, high crystallinity, and excellent photocatalytic performance was obtained by annealing at 400 °C. We also found that the use of higher annealing temperatures resulted in a reduction in the catalytic performance because of the overgrowth of the microspheres. In contrast, the photocatalyst prepared at low temperatures contained unreacted organic compounds, which reduced the photocatalytic efficiency. In addition, a detailed mechanism for the photocatalytic degradation process is proposed.

## 2. Experimental Section

### 2.1. Materials

Zinc acetate (99%), urea (99.5%), and citric acid monohydrate (99.8%) were purchased from Aladdin Biochemical Technology Co., Ltd. (Shanghai, China). All chemical reagents were analytically pure and used without further purification. A Millipore Milli-Q water purification system was used to produce ultrapure deionized water.

### 2.2. Synthesis

First, the hierarchical ZnO microsphere precursor was prepared using a hydrothermal method. Briefly, zinc acetate (0.5 g), urea (0.45 g), and citric acid (0.11 g) were added to deionized water (65 mL), and the solution was stirred magnetically for 60 min until a clear solution containing no undissolved solids was obtained. This solution was then placed in a 100 mL hydrothermal reactor and placed in a blast drying oven (DZF-6030A, Anhui Bei Keke Equipment Technology Co., Ltd. Hefei, China) at 150 °C for 3 h. After the reaction had completed, the reactor was left to cool to room temperature. The reaction products were then extracted, filtered, washed, and dried to obtain the hierarchical ZnO microspheres precursor. Subsequently, the precursor was annealed in air in a tubular sintering furnace (BTF-1200C, Anhui Bei Keke Equipment Technology Co., Ltd. Hefei, China). To find the optimal annealing temperature regarding photocatalytic performance, annealing at temperatures of 200, 300, 350, 400, 450, and 500 °C was carried out, and these temperatures were approached using a heating ramp rate of 5 °C·min^−1^. Once the target temperature had been reached, the sample was maintained at this temperature for 2 h.

### 2.3. Characterization

The crystallinity of the hierarchical ZnO microspheres was evaluated using X-ray diffractometry (XRD, Rigaku Inc., Japan) measurements on a Rigaku D/MAX-RB powder diffractometer. For these measurements, CuKα X-rays (λ = 1.5418 Å) generated at 40 kV and 60 mA were used. The XRD patterns were collected from 20 to 80° in 2θ with a step size of 0.02°. The interplanar distances for the (002) and (100) Miller planes, *d*_(002)_, and *d*_(100)_, respectively, can be calculated using Bragg’s formula [26], 2 *d*sin*θ* *= λ*, where λ is the X-ray wavelength (0.15418 nm), and *θ* is the angle between the incident ray and the scattering plane. Further, the relationship between the interplanar spacing and Miller index (*h k l*) for hexagonal structures is given by Equation (1)

(1) $$d_{hkl}={\left(\frac{4}{3}\frac{h^{2}+hk+k^{2}}{a^{2}}+\frac{l^{2}}{c^{2}}\right)}^{-\frac{1}{2}}$$

Therefore, *c* = 2 *d*_(002)_ and *a* = 2 *d*_(100)_/sqrt(3). In addition, the size of the crystallites (*D*) in the samples were calculated using the Scherrer formula (Equation (2)) [27]:

(2) $$D=\frac{0.9\lambda }{\beta \cos\theta }$$

Here, *β* is the full width at half maximum (FWHM) of the (002) reflection, and λ is the X-ray wavelength (0.15418 nm).

In addition, the thermal stabilities of the samples were determined by thermogravimetric analysis (TGA, Thermo plus EVO2, Rigaku Inc., Japan), and the sample morphologies were analyzed by field-emission electron microscopy (FE-SEM, Gemini 500, Zeiss, Germany). The chemical composition of the hierarchical ZnO microspheres and electronic states of the surface atoms were determined using X-ray photoelectron spectroscopy measurements (XPS, PHI 5000 VersaProbe, Japan) with a monochromatized Al-Kα source. Transmission electron microscopy (TEM, FEITecnai G2 F30, USA) measurements to observe the microstructures of the nanoparticles were carried out at an accelerating voltage of 200 kV. For these measurements, the samples were prepared as an ethanol suspension by ultrasonication for 10 min, then each sample was dropped on a copper mesh. The nitrogen adsorption desorption isotherms of the samples were obtained using a Micromeritics ASAP surface area analyzer. The UV-visible absorption spectra were obtained at room temperature using a UH4150 spectrometer (Hitachi Inc., Japan).

The photodegradation of the organic dyes in the presence of hierarchical ZnO microspheres and light irradiation was carried out using a PXC50C photocatalytic reactor (Beijing Perfectlight, Beijing, China). The light source was a 365 nm light-emitting diode (LED) with an areal power density of 280 mW·cm^−2^. As mentioned, the organic dyes RhB and MB were selected as model pollutants for degradation. For the photocatalytic degradation experiments, 50 mg of hierarchical ZnO microspheres was added to an aqueous solution of the organic dye (50 mL, 10 mg·L^−1^) and ultrasonicated for 10 min. Then, the solution was stirred for 1 h to ensure that adsorption-desorption equilibrium has been reached. Before illumination, 3 mL of the reaction solution was extracted for analysis. Once irradiation had started, 3 mL samples were extracted periodically for analysis. These aliquots were centrifuged, and the supernatant was quantitatively analyzed by UH4150 spectrometer (Hitachi Inc., Japan) to determine the concentration of the organic dye.

Photocurrent measurements of the samples were performed on a electrochemical workstation (CHI660, Chenhua Inc., Shanghai, China) with a three-electrode system. For these measurements, photocatalyst powder (10 mg) was dispersed in ethanol (1 mL), and Nafion solution (50 μL) was added. A uniform suspension was achieved by ultrasonication for 30 min. For transient photocurrent tests, the suspension (150 μL) was dropped onto indium-doped tin oxide glass and dried at room temperature; this was used as the working electrode. The reference and counter electrodes were Ag/AgCl and Pb electrodes, respectively. The electrolyte was 0.5M Na_2_SO_4_ solution.

Radical trapping experiments were carried out using a Bruker EMXplus electron paramagnetic resonance (EPR) spectrometer to identify the hydroxyl (·OH), and superoxide (O_2_^−^) radicals. For these experiments, 5,5-dimethyl-1-pyridine-N-oxide (DMPO) was used as the radical trapping agent, and the ZnO catalysts were dispersed in deionized water or CH_3_OH.

## 3. Results and Discussions

**Figure 1:**
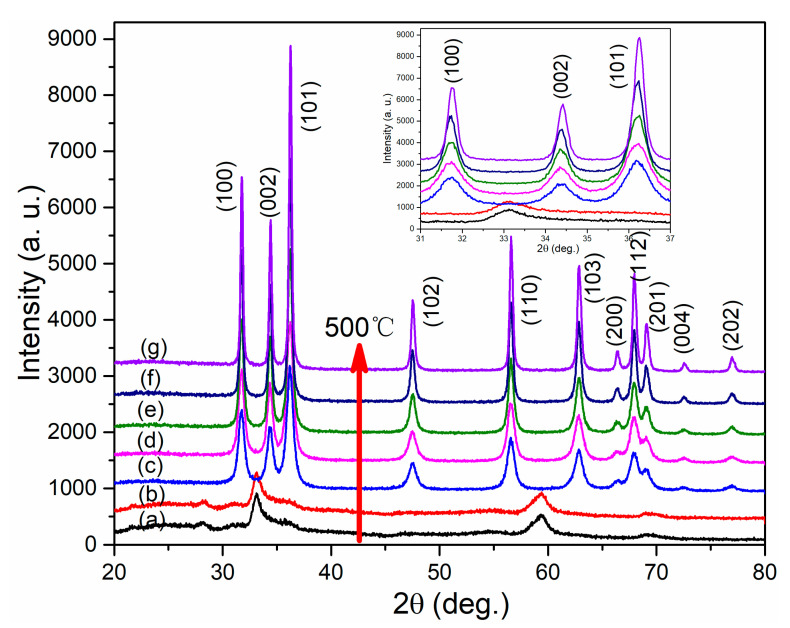
XRD patterns of hierarchical ZnO microspheres prepared at (**a**) RT, and annealed at (**b**) 200, (**c**) 300, (**d**) 350, (**e**) 400, (**f**) 450, and (**g**) 500 °C. The inset shows an enlargement of the 31~37° region.

The XRD patterns of the samples annealed at different temperatures are shown in Figure 1. For the sample annealed at 200 °C, only three significant reflections at 33.1, 59.4, and 69.4° were observed. In contrast, the XRD patterns of the samples annealed at 300 °C or higher contained different peaks. By comparison with JCPDS card No. 25–1029, we confirmed that these diffraction peaks belong to zinc hydroxide. Zinc hydroxide is formed because zinc acetate is hydrolyzed during the hydrothermal treatment, but this is then converted to the ZnO phase by high temperature calcination. For the sample annealed at 300 °C, eleven diffraction peaks appear in the XRD pattern, consistent with hexagonal wurtzite ZnO (JCPDS card No. 65–3411) [28], as shown in Figure 1.

The intensities of these diffraction peaks gradually increased with increase in annealing temperature, and a magnified view of the major diffraction peaks, (100), (002), and (101), is shown in Figure 1. As shown, the (002) reflection gradually shifts to higher angles with increase in annealing temperature, and the corresponding lattice parameters are listed in Table 1. In particular, the *c* axis is larger than that of standard ZnO powder but approaches that of the standard sample with increase in annealing temperature. Moreover, the crystallite size also increased with increase in annealing temperature. Therefore, we can conclude that the crystallinity of the hierarchical ZnO microspheres was gradually improved with increase in the annealing temperature, probably because the organic precursor was broken down and the small crystallites bonded to each other and grew at high temperatures.

**Figure 2:**
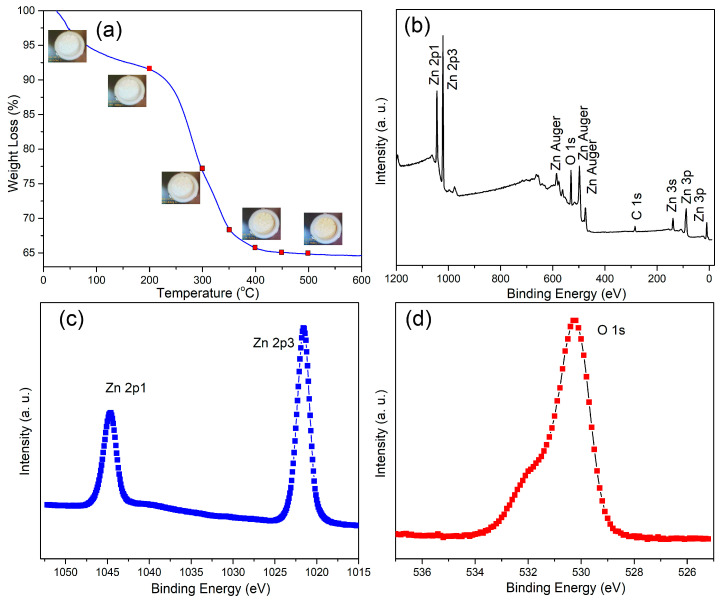
(**a**) TGA curves, (**b**) XPS survey spectra, and (**c**) Zn 2p and (**d**) O1s core-level spectra of hierarchical ZnO micro-spheres annealed at 400 °C.

To investigate the changes in mass of in the hierarchical ZnO microspheres during annealing, TGA measurements were carried out (Figure 2a). As shown in Figure 2a, for the catalyst annealed at 200 °C, there was only about 8% mass loss, and this can be attributed to the loss of crystal water. For this sample, the ZnO phase was not formed, as indicated by XRD analysis. However, at annealing temperatures above 450 °C, the mass loss was close to 35%. The decomposition of organic compounds in the sample into gaseous components is main cause of mass loss. Subsequently, the increase of annealing temperature did not result in mass loss. In addition, XPS analysis of sample annealed at 400 °C, i.e., the optimal sample, was carried out. The survey spectrum in Figure 2b shows the presence of Zn, O, and C. The C is a result of contamination with carbon from the environment during annealing and was used for binding energy calibration. As shown in Figure 2c, two peaks at 1021.5 and 1044.6 eV were observed, corresponding to Zn 2p_3/2_ and Zn 2p_1/2_ [29], respectively. As shown in Figure 2d, the O1s core-level spectrum contains peaks consistent with O in ZnO. Combining these results with those of XRD analysis, we can determine that the crystallinity of the sample is closely related to the annealing temperature. Specifically, the samples do not form the hexagonal wurtzite ZnO phase at low annealing temperatures, and higher annealing temperature are required to achieve this transition.

**Figure 3:**
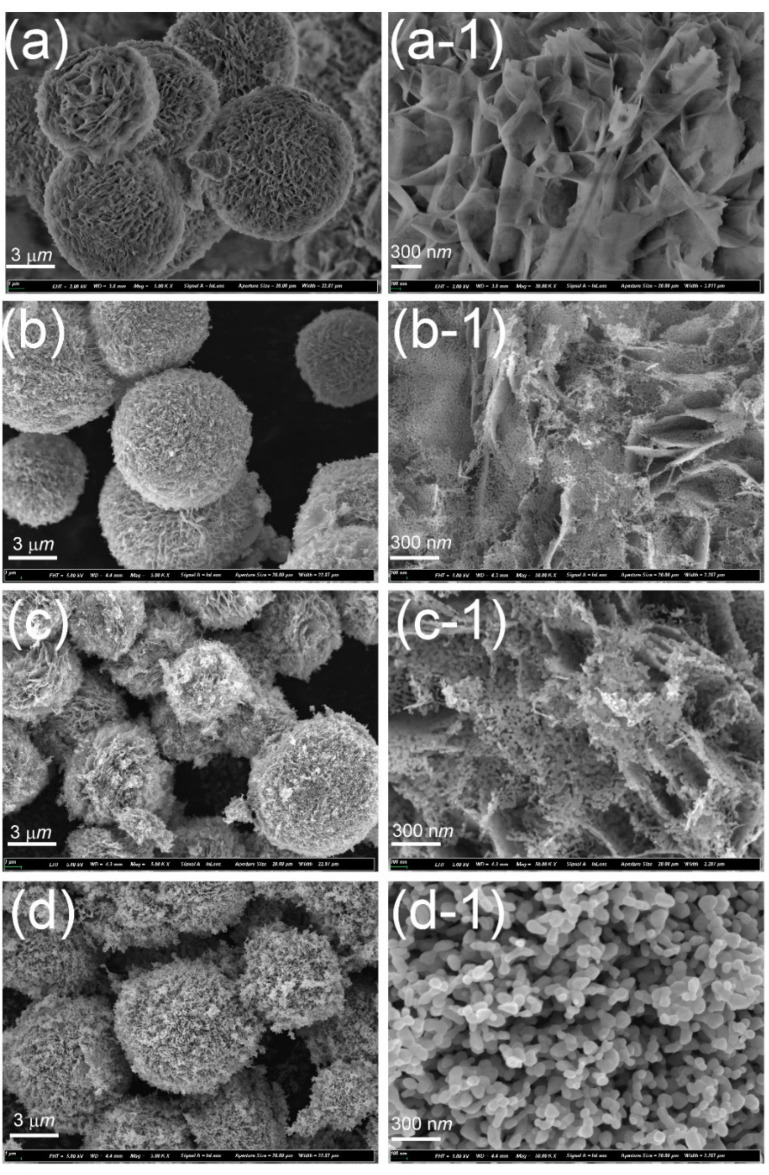
SEM images of the hierarchical ZnO microspheres prepared at (**a**) RT and annealed at (**b**) 300, (**c**) 400, and (**d**) 500 °C. (**a-1**), (**b-1**), (**c-1**), and (**d-1**) show the corresponding high-resolution SEM images.

**Figure 4:**
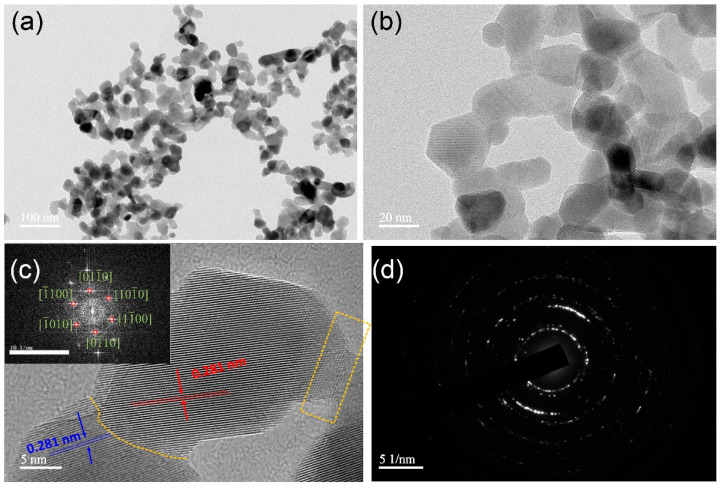
(**a**,**b**) Low-magnification TEM images, (**c**) high-resolution TEM image, and (**d**) selected area electron diffraction pattern of hierarchical ZnO microspheres prepared at an annealing temperature of 400 °C. The inset in (**c**) shows the corresponding Fourier transform of the HRTEM image.

The morphologies of the hierarchical ZnO microspheres prepared by the hydrothermal method before and after annealing are shown in Figure 3. As for the as-deposited samples (Figure 3a,a-1), the particles are spheres of about 8–10 μm in size, and each microsphere is composed of complex interlaced hierarchical nanosheets. For the sample annealed at 300 °C (Figure 3b,b-1), micro sized spheres were also formed, but the hierarchical structure gradually evolved into nanoparticles of around 15 nm connected into a network. With further increase in the annealing temperature to 400 °C, the nanoparticles formed a network structure, and grew to more than 20 nm in size, as shown in Figure 3c,c-1. After annealing at 500 °C, the size of the nanoparticles increased to about 35 nm, as shown in Figure 3d,d-1. Notably, the sizes of the nanoparticles in the hierarchical structure increased with increase in the annealing temperature, as also observed in the XRD measurements. To understand the microscopic structure and obtain the crystal lattice spacings of the hierarchical structure prepared by annealing at 400 °C, TEM measurements were conducted, as shown in Figure 4.

As shown by Figure 4a,b the hierarchical nanomaterials are composed of two-dimensional nanosheets approximately 20 nm in length, and a two-dimensional network is formed between the nanosheets. In addition, the surfaces of the nanosheets are smooth and their thicknesses is uniform. The HRTEM image in Figure 4c confirms that the nanosheets are single crystals, and the lattice fringes correspond to a d-spacing of 0.281 nm, consistent with that of single-crystal wurtzite ZnO, and indicate growth along [100] [30]. The fast Fourier transform of the HRTEM image is shown in the inset of Figure 4c and is consistent with this conclusion. In addition, the selected area electron diffraction (SAED) pattern (Figure 4d) contains diffuse rings and regular spots, adding further weight to this conclusion. Furthermore, the HRTEM images indicate that the ZnO nanosheets are not independent but are connected to each other by dislocation.

**Figure 5:**
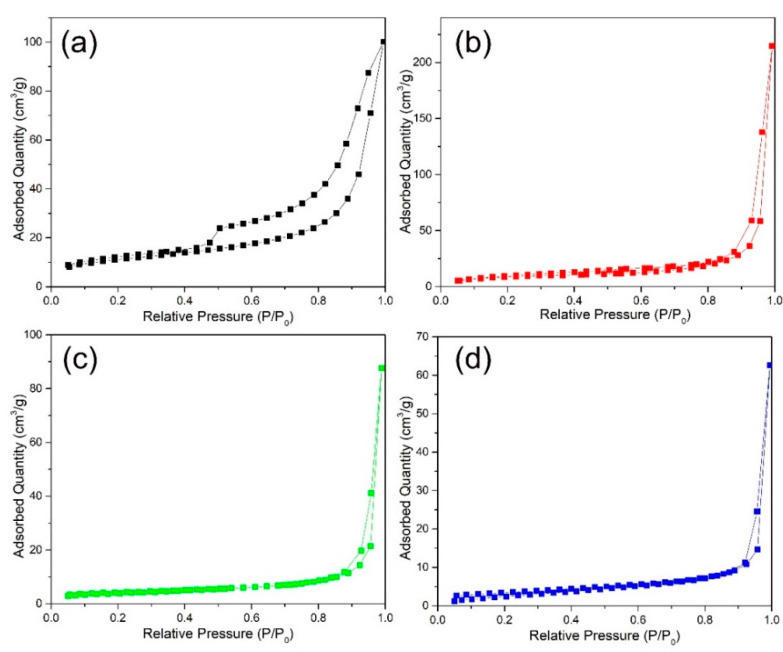
N_2_ adsorption desorption isotherms of hierarchical ZnO microspheres prepared at (**a**) RT and annealed at (**b**) 300, (**c**) 400, and (**d**) 500 °C.

Figure 5 shows the N_2_ adsorption desorption isotherms of hierarchical ZnO microspheres. The Brunauer–Emmett–Teller specific surface areas of the unannealed sample and those annealed at 300, 400, and 500 °C are 33.9, 24.4, 14.9, and 12.3 m^2^g^−1^, respectively, and the corresponding average pore sizes obtained from the pore size obtained from the pore size distributions are 18.2, 54.6, 36.5, and 31.4 nm, respectively. The specific surface area decreases because of decomposition of the organic compounds with increase in annealing temperature, whereas the pore size increases first and then decreases. The development of the layered ZnO into a network of nanoparticles resulted in an increase in the pore size during annealing. Further, at high temperatures, the size of the nanoparticles in the network gradually increases, thus reducing the size of the pores.

**Figure 6:**
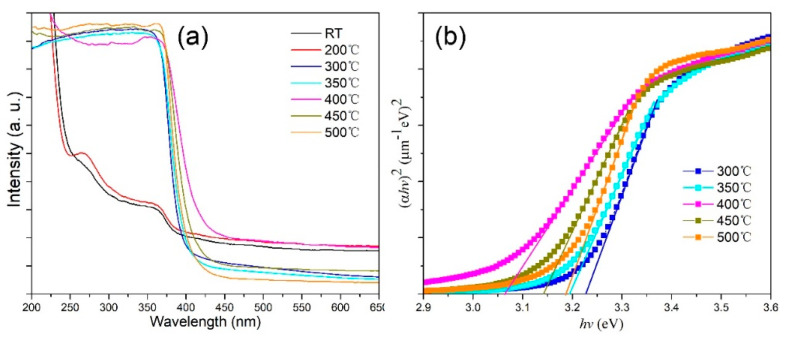
(**a**) UV-visible absorption spectra and (**b**) plots of (*αhv*)^2^ vs. *hv* for the hierarchical ZnO microspheres prepared at different annealing temperatures.

The optical band gaps of the hierarchical ZnO microspheres annealed at different temperatures were also characterized using diffuse reflectance spectroscopy measurements (Figure 6a). The absorption spectra of the unannealed hierarchical ZnO microspheres and those annealed at 200 °C are different from those of other samples because ZnO crystals were not formed at low annealing temperatures; this is consistent with the XRD and TGA results. The samples annealed at temperatures higher than 300 °C exhibited a distinct absorption edge at about 400 nm. The optical band gap (Eg) of the hierarchical ZnO microspheres can be fitted using the Tauc formula: $\alpha hv=A{\left(hv-E_{g}\right)}^{n}$ [31], where α, A, and h are the absorption coefficient, a constant, and Planck’s constant, respectively. Here, n is 1/2 for the ZnO phase. The relationship between (*αhv*)^2^ and *hv* is plotted in Figure 6b, and the intersection of the linear extension of the absorption edge and the horizontal axis gives the optical band gap (see Table 1). As shown, *E*_g_ decreased initially and subsequently increased with increase in annealing temperature. The decrease in E_g_ is a result of the increasing crystallinity, whereas the increase in E_g_ is a result of the increase in the size of the nanosheets.

**Figure 7:**
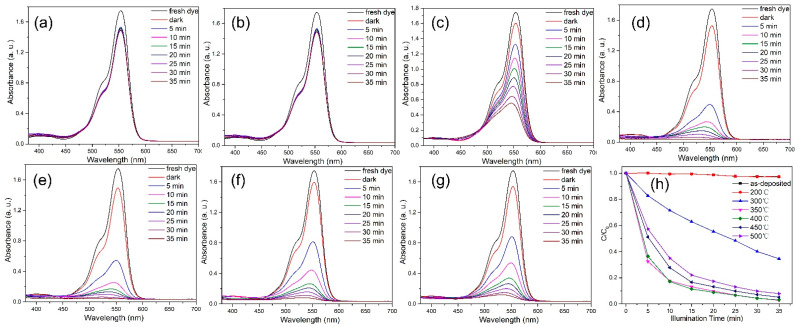
UV-vis absorbance spectra of RhB solutions treated with the ZnO micro-sphere catalysts prepared at (**a**) RT and annealed at (**b**) 200, (**c**) 300, (**d**) 350, (e) 400, (**f**) 450, and (**g**) 500 °C. The samples were illuminated for different periods. (**h**) Photodegradation of RhB catalyzed by the hierarchical ZnO micro-spheres annealed at different temperatures.

To evaluate the photocatalytic ability of the catalysts for the degradation of RhB and MB under light irradiation, the remaining dye concentrations were calculated, as shown in Figure 7a–g. The degradation efficiency was calculated as (1 − C/C_0_) × 100%, where C_0_ and C represent the dye concentration at absorption equilibrium in the dark and after light exposure, respectively [32], and the relationship between C/C_0_ and illumination time is plotted in Figure 7h. Figure 7 shows that the physical adsorption of dyes on all catalysts was similar. However, the catalyst prepared at low annealing temperatures had no photocatalytic degradation ability. In contrast, the catalysts annealed at 300 °C and above degraded RhB on UV irradiation, and the best activity was obtained for the catalyst annealed at 400 °C. Subsequently, with further increase in the annealing temperature, the photodegradation ability decreased.

**Figure 8:**
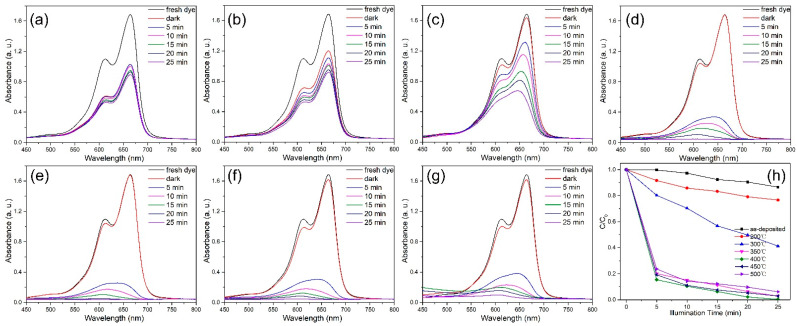
UV-vis absorbance spectra of MB solutions treated with the photocatalyst prepared at (**a**) RT and annealed at different temperatures for different periods: (**b**) 200, (**c**) 300, (**d**) 350, (**e**) 400, (**f**) 450, and (**g**) 500 °C. (**h**) Degree of photodegradation of MB annealed at different temperatures.

Figure 8a–g shows the UV-vis absorption spectra obtained during the degradation of MB by the catalyst during photodegradation. Plots of C/C_0_ versus time are shown in Figure 8h. As shown, the best degradation performance was obtained for the catalyst annealed at 400 °C, as for the degradation of RhB. However, MB was degraded more rapidly than RhB by the photocatalyst.

**Figure 9:**
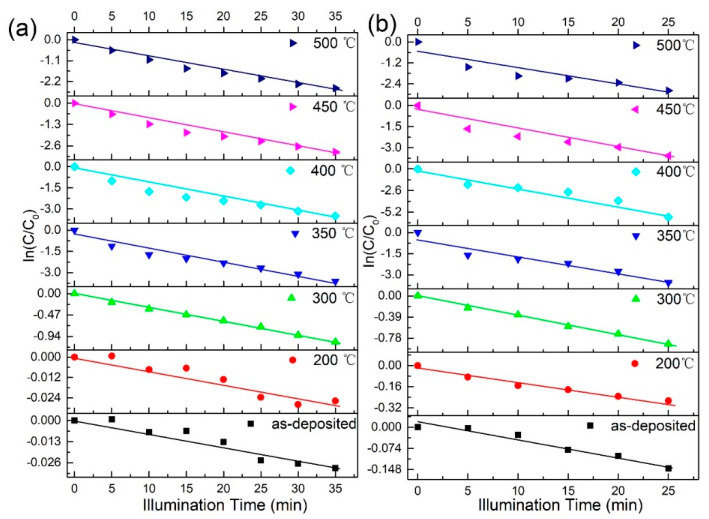
Kinetic plots of the photodegradation of (**a**) RhB and (**b**) MB by the photocatalysts prepared at different temperatures.

Moreover, the photodegradation reaction kinetics follow the pseudo-first-order kinetics equation [33]: In (*C*/*C*_0_) = −*kt*, where k and t denote the degradation rate constant and reaction time, respectively. The kinetic fits to the experimental data are shown in Figure 9. The fit is reasonably linear, thus, the photodegradation rates, *k*_1_ and *k*_2_, for RhB and MB were obtained from the gradients of the fits to the data (see Table 1). The fastest rates, 0.1021 and 0.217 for RhB and MB, respectively, were obtained for the catalyst annealed at 400 °C. As shown, the degradation rate of MB is twice that of RhB.

**Figure 10:**
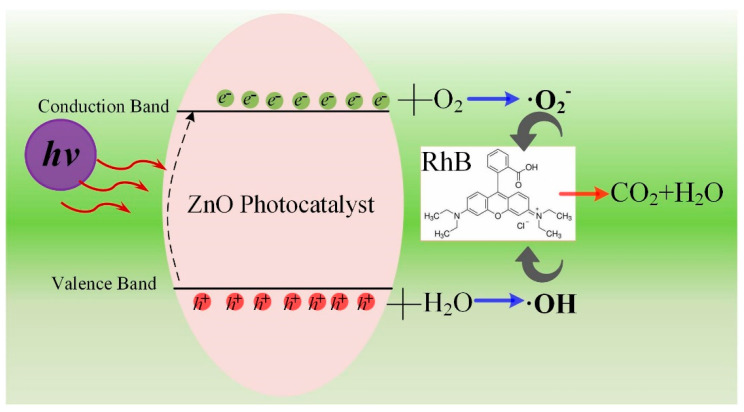
Schematic of the mechanism of photodegradation of organic dyes by the hierarchical ZnO microsphere catalyst.

Next, the mechanism of degradation of the organic dyes by the catalyst was analyzed; the proposed mechanism is shown in Figure 10. Briefly, when UV light hits the catalyst, it excites electrons from the valence band into the conduction band, resulting in the formation of holes in the valence band. The electrons in the conduction band move to the surface of the catalyst where they can react with oxygen dissolved in the solution to form·O_2_^−^. Meanwhile, the holes in the valence band react with water to form OH. The OH and O_2_^−^ act simultaneously on organic dyes to mineralize them. Thus, O_2_^−^ and ·OH play a crucial role in degrading the dyes, and their concentration determines the rate of degradation.

**Figure 11:**
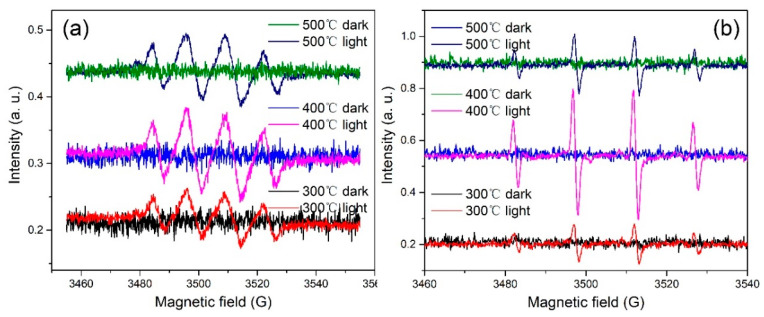
EPR spectra of the hierarchical ZnO microsphere catalysts annealed at 300, 400, and 500 °C in the dark and under light irradiation: (**a**) DMPO-O_2_^−^ and (**b**) DMPO-OH.

To verify the mechanism proposed above, EPR spectra of the catalysts in the dark and under illumination were collected, as shown in Figure 11. No EPR signals were observed in the dark. In contrast, under illumination, signals corresponding to DMPO-·OH and DMPO-O_2_^−^ were observed, and the EPR signal intensity of the catalyst annealed at 400 °C was the strongest, indicating that the sample generated the most DMPO-·OH and DMPO-O_2_^−^ on light irradiation. These results are consistent with the proposed photodegradation mechanism.

**Figure 12:**
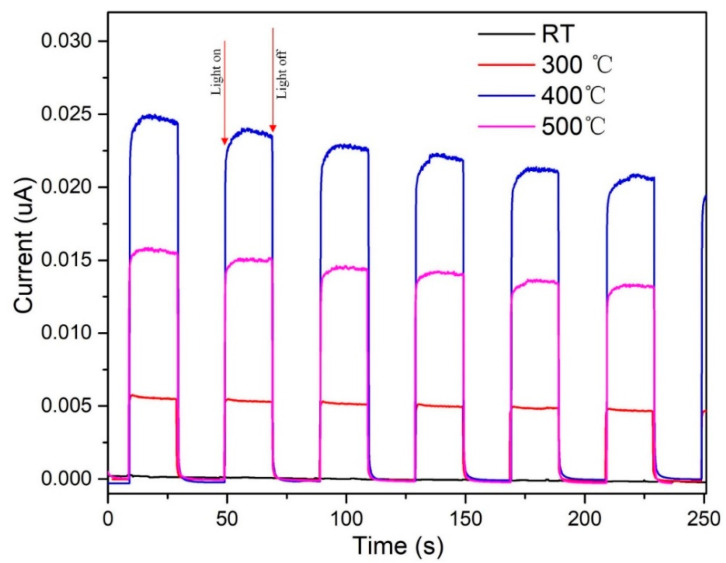
Transient photocurrents of the as-deposited hierarchical ZnO microspheres and hierarchical ZnO micro-spheres annealed at 300, 400, and 500 °C after exposure to UV light.

In addition, the separation and transfer dynamics of the photoexcited charge carriers were investigated using transient photocurrent measurements with turn-on-turn-off cycles (Figure 12). Clearly, light irradiation was crucial for the degradation process, as shown by the lack of photocurrent in the dark. In addition, when the light was turned on, the transient photocurrent density of the ZnO catalyst annealed at 400 °C was far higher than those of the catalysts prepared at other annealing temperatures, meaning that the photoinduced charge carriers in the hierarchical ZnO microspheres were effectively separated, leading to a relatively low recombination rate.

Further, the samples prepared at low annealing temperatures comprise hierarchical ZnO microspheres having a cubic structure with low crystallinity, which has almost no photodegradation performance. With an increase in the annealing temperature, the hierarchical ZnO microspheres gradually crystallized into the hexagonal phase, and the crystallinity improved. From the SEM images, it can be seen that the microspheres had a two-dimensional sheet structure, and the uniform thickness of the two-dimensional nanosheet structure was conducive to the separation of photogenerated carriers, which improves the photodegradation performance of the catalyst. However, when the annealing temperature was further increased to more than 450 °C, a nano-flake structure formed and the specific surface area and pore size decreased, leading to a reduction in the photodegradation performance.

## 4. Conclusions

In this study, a hierarchical ZnO microsphere precursor was fabricated by a hydrothermal method, and the catalyst crystallinity and morphology were controlled by varying the annealing temperature. When the annealing temperature was greater than 300 °C, the ZnO microspheres exhibited a hexagonal wurtzite structure, and the crystallinity was high. The hierarchical ZnO microspheres were approximately 8–10 μm in size and composed of complex interlaced hierarchical nanosheets. In addition, a two-dimensional network was formed between the nanosheets. Crucially, the surfaces of the nanosheets were smooth, and the thickness of the nanosheets was uniform. Notably, the ZnO nanosheets were not independent but connected to each other by dislocation. The photodegradation tests showed that the annealing temperature had an important effect on the catalytic activity, and the sample annealed at 400 °C showed excellent degradation performance. Radical trapping revealed the formation of·OH and O_2_^−^ free radicals on light irradiation, which facilitated the breakdown of organic dyes, and the EPR signal strength of the hierarchical ZnO microspheres annealed at 400 °C was the strongest, indicating that the sample generated the most OH and·O_2_^−^ radicals on irradiation. Finally, the transient photocurrent density was far higher than those of the other catalysts annealed at different temperatures, meaning that the photoinduced charge carriers in the hierarchical ZnO microspheres were effectively separated. These findings provide support for the proposed photodegradation mechanism. Thus, we have demonstrated a simple method for the production of a highly active photocatalyst, and our findings concerning the effect of the annealing temperature, which could aid in the production of other similar active photocatalysts for wastewater remediation and other photocatalytic applications.

## Figures and Tables

**Table 1:** Unit cell parameters calculated from the XRD patterns, optical band gaps, and photodegradation rates of the hierarchical ZnO micro-spheres prepared at different annealing temperatures.

Sample	(h k L) *^a^*	2*θ* *^b^* (^o^)	d_hkl_ *^c^* (nm)	a *^d^* (nm)	c *^e^* (nm)	β_002_ *^f^* (^o^)	D *^g^* (nm)	*E_g_^h^*	*k*_1_ *^k^* (min^−1^)	*k*_2_ *^l^* (min^−1^)
RT	—	—	—	—	—	—	—	—	0.0008	0.0051
200 °C	—	—	—	—	—	—	—	—	0.0008	0.011
300 °C	(002)	34.35	0.2610	0.3259	0.5221	0.59	14.1	3.23	0.0301	0.036
	(100)	31.70	0.2823							
350 °C	(002)	34.36	0.2609	0.3258	0.5219	0.56	14.9	3.19	0.1016	0.1201
	(100)	31.71	0.2821							
400 °C	(002)	34.37	0.2609	0.3257	0.5218	0.45	18.4	3.06	0.1021	0.2172
	(100)	31.72	0.2820							
450 °C	(002)	34.38	0.2608	0.3256	0.5216	0.34	24.4	3.14	0.0844	0.1299
	(100)	31.73	0.2818							
500 °C	(002)	34.42	0.2605	0.3252	0.5211	0.28	29.7	3.19	0.0701	0.944
	(100)	31.77	0.2816							

*^a^* Miller index. *^b^* Bragg angle. *^c^* Interplanar spacing. *^d^* a-Axis length of unit cell. *^e^* c-Axis length of unit cell. *^f^* Full width at half maximum of indicated reflection. *^g^* Average crystallite size calculated using the Scherrer formula. *^h^* Optical band gap. *^k^* Photodegradation rate for RhB. *^l^* Photodegradation rate for MB.

## Data Availability

Not applicable.
